# Evolution characteristics and pattern optimization of land use conflict in inland river Basin from the perspective of production-living-ecological

**DOI:** 10.1371/journal.pone.0321481

**Published:** 2025-04-24

**Authors:** Wanzhuang Huang, Ziyang Wang, Yue Liu, Jing Shi

**Affiliations:** 1 College of Urban Construction, Lanzhou City University, Lanzhou, China; 2 Gansu Engineering Research Center of Land Utilization and Comprehension Consolidation, Lanzhou, China; 3 College of Geographic and Environmental Science, Northwest Normal University, Lanzhou, China; Agriculture and Forestry University, NEPAL

## Abstract

Identification of the process and intrinsic mechanisms of land use conflicts is a prerequisite for adjusting optimal national spatial planning strategies. This paper, starting from the perspective of production-living-ecological, constructed a train of thought for optimizing national spatial planning in inland river basins, taking the example of the Shiyang River Basin. Based on the conflict measurement model of “source of risk - receptor of risk - effect of risk”, this paper analyzed the evolution of production-living-ecological land conflicts, employed a geographical weighted regression model to examine the influencing factors of the evolution of land use conflicts, and optimized the national spatial pattern as well as proposed regulation strategies. The results indicated that from 1990 to 2020, the area of unused land in the Shiyang River Basin decreased the most, mainly converted into farmland, construction land, and water areas. The transfer of land use mainly manifested as mutual transformation between production land and ecological land, followed by mutual transformation between production land and living land. During the research period, the overall complexity of land use presented a pattern of “high in the south and low in the north”, the overall vulnerability showed a pattern of “high in the central area and low in the surrounding areas”, and the overall stability was relatively high. The intensity of production-living-ecological land conflicts in the Shiyang River Basin showed an increasing trend, with the average conflict intensity rising from 0.44 to 0.57, exhibiting a spatial distribution pattern of “high in the central area and low in the surrounding areas”. The impact of road network density and river network density on the evolution of production-living-ecological land conflicts in the Shiyang River Basin was the most significant. The optimization results of national spatial patterns and regulation strategies could provide a scientific basis for the optimization and comprehensive management of national spatial planning in arid inland river basins.

## Introduction

The territorial space provides a multitude of functions and services for humanity and serves as the primary locus for human life and production. With rapid industrialization and urbanization, unprecedented impacts and challenges have emerged regarding the rational layout and development of territorial space. Sustainable development of territorial space faces formidable challenges and crises [1,2]. The industrialization and urbanization processes in inland river basins are accelerating, leading to the increasing land demand for human production and living. As a result, ecological spaces are being encroached upon, and the limited land supply has led to an imbalance in the structure of production, living, and ecological land. This exacerbates the conflicts and contradictions among production, living, and ecological land [3]. Therefore, the scientific identification of areas for production-living-ecological purposes, coupled with an exploration of their spatiotemporal evolution patterns, serves as the foundation and prerequisite for context-specific prevention and resolution of land use conflicts. Moreover, the divergent development goals of different regions, influenced by varying geographical and developmental conditions, contribute to conflicting ideologies such as prioritizing economic development at the expense of ecological considerations [4,5]. There is an urgent need to explore the relationship between social, economic, and natural factors and the response to land use conflicts. This research aims to reconcile the contradictions between socioeconomic development and ecological preservation, thereby formulating territory spatial regulation strategies conducive to achieving regional development objectives.

The multifunctionality of national territory spatial planning forms the foundation for the generation of land use conflicts. The primary cause of land use conflicts phenomena lies in the competition for scarce land resources resulting from different land-use patterns in the same region. The essence of land use conflicts issues lies in the lack of coordination between the current allocation of land resources and the sustainable development of landscapes [6,7]. Current academic research on land use conflicts primarily encompasses the mechanisms of formation, identification of types, hierarchical assessment, evolutionary characteristics, impact effects, and regulatory management [8–10]. Research methods mainly include participatory interviews, suitability assessments, PSR models, multi-criteria evaluation, coupled coordination assessment, and landscape ecological risk analysis [11–13]. However, most studies utilize administrative units as evaluation units, making it difficult to accurately reflect the distribution of land use conflictss and spatial adjacency effects [14,15]. The greater the ecological risks resulting from land use evolution, the higher the intensity of conflicts they tend to trigger. This paper employs a conceptual model of landscape pattern and ecological risk assessment [16], utilizing the “external pressure + vulnerability - stability” metrics derived from landscape indices to characterize the origins, receptors, and ramifications of landscape ecological risks. Building upon this framework, a quantitative method for assessing land use conflicts is devised to achieve a comprehensive understanding of their extent and nature. This approach not only delineates spatial landscape patterns but also exposes the regional ecological hazards stemming from irrational land use configurations. Furthermore, it precisely identifies the location and intensity of land use conflicts at the grid level, furnishing a scientific underpinning for the development of a national spatial planning system. It aids in finely regulating and directing human development activities, scientifically discerning land use conflicts, and facilitating targeted mediation, thereby playing a pivotal role in shaping the framework for land spatial protection and development.

The root cause of land use conflicts lies in the regional discordance and imbalanced configuration of land use structure and territorial spatial functions. Areas designated for productive-living-ecological purposes not only reflect land use patterns but also embody regional territorial spatial functions. Therefore, examining land use conflicts through the lenses of producti, living and ecology can, to some extent, provide a microcosm and comprehensive insight into all conflicts [17]. This approach facilitates the accurate and comprehensive identification of critical conflict zones and enables the proposition of more targeted optimization strategies for territorial spatial planning. Optimizing territorial spatial patterns, an integral part of territorial spatial planning, is tasked with regulating and guiding the intensity of human development. Scientific identification and effective mediation of land use conflicts are essential pillars for establishing a framework for territorial spatial protection and development. Presently, scholars all over the world are actively involved in research and practical initiatives focused on optimizing territorial spatial patterns, particularly in the realm of land use allocation optimization [18–20], and key research directions such as primary functional zones [21–26]. Substantial advancements have been achieved in theoretical underpinnings, empirical investigations, and research methodologies [27–31]. However, in the course of optimizing territorial spatial planning, insufficient attention has been directed towards addressing land use conflicts arising from regional disparities and an imbalanced alignment between land use structure and territorial spatial functions. A clear technical pathway for transitioning from effective resolution of land use conflicts to the optimization of territorial spatial patterns is yet to be established, and practical guidance for territorial spatial optimization remains inadequately developed. Concurrently, examining the optimization of territorial spatial patterns through the lenses of production, living, and ecology facilitates the rational adjustment of production spatial layout, improvement of living space quality, and preservation of ecological space environments. This contributes to achieving harmonized and sustainable development across territorial spatial economy, society, and environment.

In arid regions, the protection of the ecological environment is particularly crucial due to the unique natural conditions of inland river basins. Against the backdrop of escalating human activity, ecological vulnerability is progressively intensifying. The conflict between human production and livelihoods and ecological conservation urgently requires alleviation [32]. Within the constraints of limited water and soil resources, how to reconcile the development of production and living land with the conservation of ecological land has emerged as a focal issue, which is constraining the sustainable development of inland river basins [33,34]. The Shiyang River Basin is situated at the confluence of the Qinghai-Tibet Plateau, Loess Plateau, and Inner Mongolia Plateau, serving as a significant oasis agricultural area and ecological barrier zone in the arid regions of northwestern China [35]. In recent years, with the socio-economic development in the Shiyang River Basin, ecological land has been encroached upon, the environment of production and living land has deteriorated, and the carrying capacity of the ecological environment has declined. Consequently, conflicts between production, living, and ecological land have become increasingly prominent, severely constraining sustainable development. Therefore, addressing the conflicts among production, living, and ecological lands and achieving coordinated development has become an urgent issue in the Shiyang River Basin. To address this issue, this paper utilizes land use data as a foundation. By constructing a classification system for production, living, and ecological land and employing a land use conflicts measurement model based on the “the risk source—the risk receptor—the risk effect” framework, it identifies the regions and intensities of land use conflicts. Additionally, it utilizes a geographic weighted regression model to explore the factors influencing the evolution of conflicts among production, living, and ecological land. Finally, this paper proposes optimization strategies for the protection and development of territorial space and suggests regulatory measures, aiming to provide scientific guidance for the construction of territorial space development and ecological environment protection in the Shiyang River Basin.

## Materials and methods

### Study area

**Fig 1:**
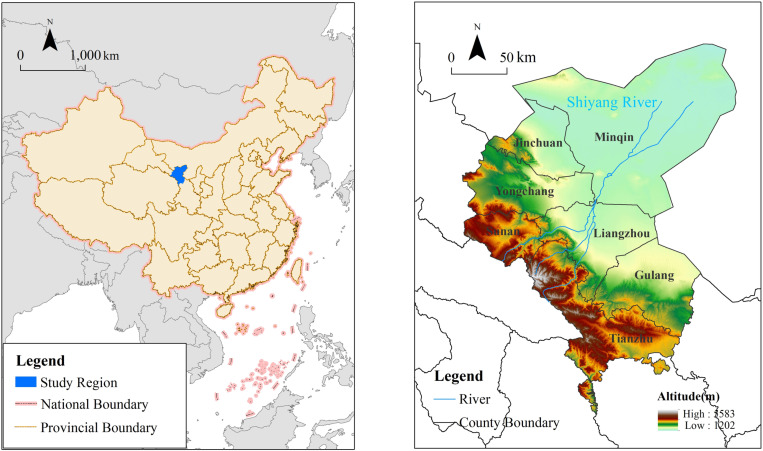
Geographical setting of Shiyang River basin. (The map was generated by ArcGIS 10.4 http://www.esri.com/software/arcgis and does not require any permission from anywhere).

The Shiyang River Basin is located at the intersection of the three major plateaus: Loess Plateau, Qinghai-Tibet Plateau, and Inner Mongolia-Xinjiang Plateau(Fig 1). It is situated in the eastern part of the Hexi Corridor in Gansu Province, with geographical coordinates ranging from 101°41′ to 104°16′E and 36°29′ to 39°27′N. The basin includes Liangzhou District of Wuwei City, Gulang County, and Minqin County, as well as parts of Yongchang County, Jinchang District, and Sunan Yugur Autonomous County in Jinchang City. The terrain is characterized by the southern Qilian Mountains region, the central plain area, and the northern desert region. The basin is located deep in the continental interior and experiences a continental temperate arid climate. The average annual temperature in the basin is 6.4°C, with an annual precipitation of 260.0 mm. It is a typical inland river basin in an arid region, with a higher elevation in the south and lower elevation in the north. Overall, the basin can be divided into four major geomorphic units: the southern Qilian mountainous area, the central corridor plain area, the northern low mountain and hill area, and the desert area. The basin has a resident population of 1.9 million people, making it one of the most densely populated, highly developed in terms of water and soil resources, and environmentally challenged river basins in China’s inland river systems [36].

### Data source

**Fig 2:**
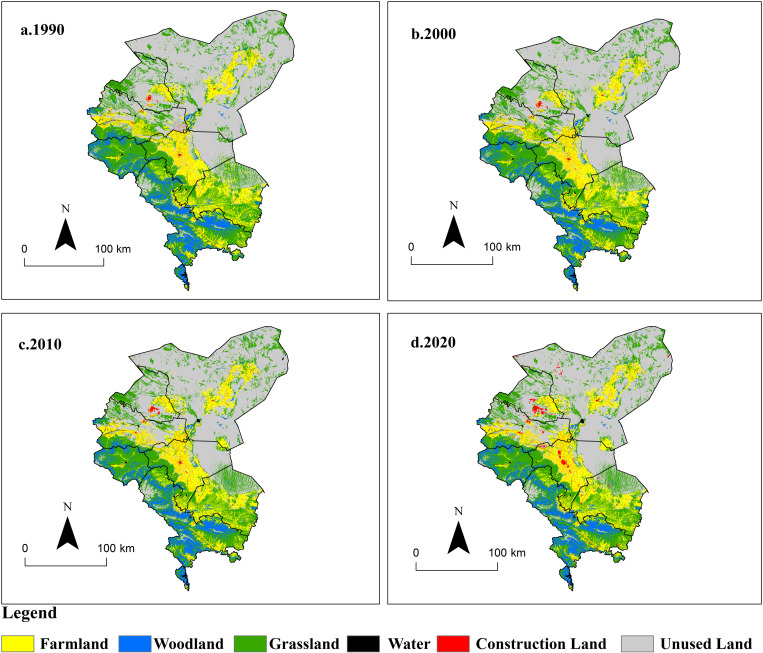
Land use change of Shiyang River from 1990 to 2020. (The map was generated by ArcGIS 10.4 http://www.esri.com/software/arcgis and does not require any permission from anywhere).

The data used in this study mainly include basic geographical data (administrative boundaries, hydrology), Digital Elevation Model (DEM), land use/land cover change (LUCC), population density, road network density, per capita GDP, river network density, and socio-economic statistical data related to the Shiyang River Basin. The basic geographical data (administrative boundaries, hydrology) and Digital Elevation Model (DEM) were obtained from the Chinese Academy of Sciences Resource and Environment Science Data Center (http://www.resdc.cn), with a spatial resolution of 30m. The land use data for the years 1990, 2000, 2010, and 2020 were sourced from the “China Multi-Temporal Land Use/Land Cover Remote Sensing Monitoring Database” (CNLUCC) provided by the Chinese Academy of Sciences Resource and Environment Science Data Center (http://www.resdc.cn/). The spatial resolution is 30m, and the data were interpreted through visual interpretation using a human-computer interaction method. The interpretation accuracies of the remote sensing images for the four periods were 84.6%, 85.4%, 87.3%, and 88.9%, respectively. Land use types comprise six primary categories: grassland, farmland, construction land, woodland, water, and unused land (Fig 2), as well as 25 secondary categories. Population density, road network, per capita GDP, and river data were obtained from the Chinese Academy of Sciences Resource and Environment Science Data Center (http://www.resdc.cn), with a spatial resolution of 1km. Social and economic data mainly come from the “Statistical Yearbook of Gansu Province” for the years 1991 to 2021 (http://tjj.gansu.gov.cn/). All spatial data in this study were standardized to a uniform spatial coordinate system, with a consistent resolution resampled to 1 km. By harmonizing spatial data from different sources and with varying resolutions to a 1 km resolution, the consistency and comparability of the data were enhanced, facilitating data processing and storage. This approach also contributed to the accuracy and reliability of the research findings.

### Research methods

#### Research framework.

**Fig 3:**
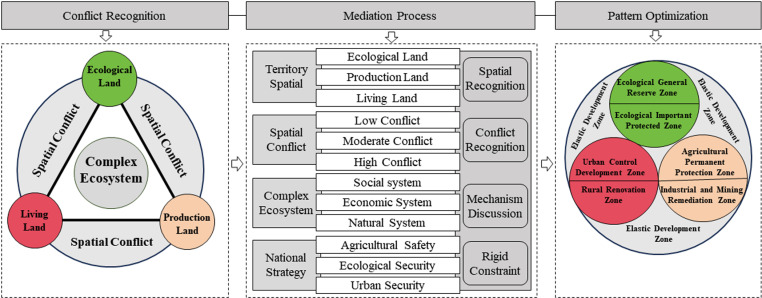
Research framework.

The watershed system constitutes a crucial component of territorial space, where surface natural phenomena and human settlement activities are intricately intertwined with watershed processes. Under the overarching principle of harmonious development between human and nature, the conflict between the scarcity of territorial resources and the inexhaustible demands of human development has further intensified the conflict between human production and living land use and ecological land [37]. The conflict between the scarcity of land and resources and the unlimited human development demands exacerbates the conflict between human production and living spaces and ecological spaces. The 18th report of the Communist Party of China proposes the national spatial management goal of “promoting intensive and efficient production space, moderately livable living space, and beautiful ecological space” [38]. Therefore, there is an urgent need to systematically construct a theoretical and technical system for identifying and optimizing the conflicts in production-living-ecological land in the inland river basin in arid regions. Based on this, this paper constructs a framework for optimizing the spatial pattern of inland river basins, including spatial identification, conflict recognition, mechanism exploration, and rigid constraints (Fig 3), and empirically studies the Shiyang River Basin. Firstly, based on dynamic degree models and land transfer matrix models, the characteristics and mechanisms of the evolution of national spatial patterns are understood. Guided by the theory of the human-land relationship geographical system, land use division is carried out from the perspectives of production, living, and ecology. Secondly, based on the concept model of “the risk source—the risk receptor—the risk effect”, an indicator system is constructed. The land use conflicts intensity of production, living, and ecological land in the Shiyang River Basin is analyzed using the land use conflict intensity index model. At the same time, from a combined perspective of social, economic, and natural factors, the intrinsic mechanisms of land use conflicts are explored based on the geographic weighted regression model. Finally, based on the current state of land use patterns, the results of land use conflict identification, the intrinsic mechanisms of conflicts, and national strategic requirements, optimization strategies for the national spatial pattern of the Shiyang River Basin are proposed.

#### Land Use Dynamics.

Land use dynamics quantitatively describe the magnitude of changes in land use, thereby revealing the intensity of a certain type of land use change in a region. The formula is as follows [39]:


$$K=\frac{S_{end}-S_{start}}{S_{start}}\times \frac{1}{T}\times 100\text{\%}$$

In the equation, K represents the dynamism of a specific land use type; S_start_ and S_end_ denote the respective areas of a certain land use type at the beginning and end of the study period. T represents the research time interval.

#### Land use transfer matrix model.

The analysis of transitions between various land use types within a study area during a specific period is typically conducted using a Land Use Transfer Matrix. This matrix not only reveals the transition directions and sources of each land use type at the end of the study period but also reflects the quantitative characteristics of these transitions. Consequently, it provides insights into the land use change patterns within the study area over the research period. By offering a more intuitive and visual representation, the matrix facilitates a clearer quantification and depiction of the interactions between ecological, productive, and living spaces in the Shiyang River Basin during a specified timeframe. The expression for this matrix is as follows [40]:


$$S=\left\{\begin{array}{l} s_{11}\cdots s_{1n}\\ s_{21}\cdots s_{2n}\\ s_{n1}\cdots s_{nn} \end{array}\right\}$$

In the equation: n represents the total number of land use types; i, j represent the land use types at the beginning and end of the study period, respectively (i, j = 1,2,…n); S_ij_ denotes the area transformed from land type i to land type j during the study period.

#### Method for identifying production, living, and ecological land.

The division of production-living-ecological land is a comprehensive zoning approach for recognizing the current land use structure, patterns, and issues. It is a crucial component of optimizing territory spatial allocation and serves as a primary basis for formulating differentiated land resource management policies. In this study, based on relevant literature and considering land use functions such as agricultural production, socio-economic development, and ecosystem services in the Shiyang River Basin [41,42], this study utilizes a land use classification method to delineate the production, living, and ecological land in the basin. Using the land use data from 1990, 2000, 2010, and 2020, which include 25 secondary land categories, the land use classification method is employed. This method, widely used at present, is primarily based on the main functions of territorial space (Table 1), enabling the delineation of the distribution pattern of production, living, and ecological land at the patch scale in the Shiyang River Basin. Given the complexity of the topography and landforms in arid inland river basins, particularly in the mountainous systems of the upper reaches of the basin, where significant variations in slope and elevation are common, the presence of gullies, canyons, and hills disrupts the continuity of land use types, making land use classification challenging. Additionally, areas where different land use categories intersect are difficult to categorize accurately, as these regions are widespread and often do not fully conform to predefined categories of land use combinations. It is evident that land use-based delineation of production, living, and ecological zones has certain limitations. However, considering the large spatial scale of this study, these limitations are deemed to have a relatively minimal impact on the overall results of the research.

**Table 1:** Classification of Production, Living, and Ecological Land.

Primary Class	Secondary Class	Definition	Subject function
Arable Land	Paddy Field	Arable land for cultivating aquatic crops	Production
	Dry Land	Arable land for cultivating non-aquatic crops	Production
Forest Land	Dense Forest	Natural or artificial forests with a canopy closure > 30%	Ecological
	Bush Forest	Shrubby forest with a canopy closure > 40% and a height below 2 meters	Ecological
	Sparse Forest	Forest land with a canopy closure of 10-30%	Ecological
	Other Forests	Economic forests, orchards, etc.	Production
Grassland	High Coverage Grassland	Natural grassland, improved grassland, or mowed grassland with a coverage > 50%	Ecological
	Moderate Coverage Grassland	Natural grassland or improved grassland with a coverage between 20%-50%	Ecological
	Low Coverage Grassland	Natural grassland with a coverage between 5%-20%	Ecological
Water Area	River	Natural or artificially excavated rivers	Ecological
	Lake	Naturally formed water bodies	Ecological
	Reservoirs and Ponds	Artificially built water storage areas	Ecological
	Perennial Glacier and Snow Areas	Areas perennially covered by glaciers and snow	Ecological
	Tidal Flat	Land between the normal water level and the maximum water level of reservoirs or ponds	Ecological
	Shoal	Land between the normal water level and the flood level of rivers or lakes	Ecological
Construction Land	Urban Land	Land used for urban areas of large, medium, and small cities as well as towns above the county level	Living
	Rural Residential Area	Residential areas independent of urban areas	Living
	Other Construction Land	Land for factories and mines, large industrial zones, oil fields, salt flats, quarries, as well as transportation roads, airports, and special purpose land	Production
Unused Land	Sandy Land	Land covered by sand with a vegetation coverage of less than 5%	Ecological
	Desert	Land mainly covered by gravel with a vegetation coverage of less than 5%	Ecological
	Saline-Alkali Land	Land with accumulated salts and alkalis, sparse vegetation, suitable only for salt and alkali-tolerant plants	Ecological
	Marsh	Flat and low-lying land with seasonal or perennial waterlogging and the surface covered with wetland plants	Ecological
	Bare Land	Land with bare soil and a vegetation coverage of less than 5%	Ecological
	Bare Rocky Land	Land surface covered with rocks or gravel	Ecological
	Others	Other unused land, including high-cold deserts, tundra, etc.	Ecological

#### The quantification model of land use conflicts.

In this study, we employed the area-weighted average fractal dimension from landscape indices to reflect the complexity of patches. A larger area-weighted average fractal dimension indicates more complex boundaries of landscape patches, suggesting a greater opportunity for interference from neighboring areas and reflecting the risk sources faced by land resources. Land use vulnerability reflects the exposure of risk receptors, with vulnerability determined by the resistance capacity of landscape patches to external disturbances. The intensity of external disturbances varies over time across different spatial types. This study draws on the approach of Zhang et al., which combines soil sensitivity in arid inland river basins with expert scoring, to obtain vulnerability factor assignments for ecological, living, and production land uses [43]. In the Shiyang River Basin, vulnerability factors are assigned values of 3, 2, and 1 for production, living, and ecological land, respectively. Land use stability is represented by patch density in the landscape index. Higher patch density indicates greater landscape fragmentation within the region, resulting in lower biodiversity and coherence, and a higher likelihood of ecological risk to land resources. Patch density negatively reflects the stability of regional landscapes, thereby representing the risk effect. Greater external disturbances experienced by land units lead to higher ecological risk exposure and lower land use stability. Consequently, the likelihood of ecological risk and land use conflicts increases. Therefore, relevant studies propose that “land use conflict = external pressure + spatial exposure - spatial stability” [44]. Finally, considering factors such as the study scope, data types, and patch conditions, we selected a spatial grid of 3km ×  3km as the assessment unit. Referring to previous research [45–47] and the conceptual model of risk sources-risk receptors-risk effects, we constructed an indicator system (Table 2) and analyzed the intensity of conflicts in production, living, and ecological land based on the land use conflicts intensity index model.

**Table 2:** Methods for Calculating Indicators of Production, Living, and Ecological Land.

Indicator Name	Computational Formula	Formula Description
Land Use Complexity Index	$\text{AWMPFD}=\overset{m}{\underset{j=1}{\sum }}\overset{n}{\underset{i=1}{\sum }}\left[\frac{2\ln\left(0.25P_{ij}\right)}{\ln a_{ij}}\times \left(\frac{a_{ij}}{A}\right)\right]$	*P*_ij_ is the perimeter of the patch; *a*_ij_ is the area of the patch; *A* is the total area of the land unit; *m* is the total number of spatial evaluation units in the study area; *n* is the total number of types of production-living-ecological land.
Land Use Vulnerability Index	$E_{i}=\sum F_{i}\times \frac{a_{i}}{A}$	*F*_i_ is the vulnerability assignment for the *i-*th type of land; *a*_i_ is the area of the *i-*th type of land.
Land Use Stability Index	$\text{S}=1-\text{PD}=1-n_{i}/\text{A}$	*PD* is patch density; *n*_i_ is the number of patches in the *i-*th type of land.
Land Use Conflict Intensity Index	$\text{LUCS}=\text{AWMPFD}＋E_{i}-S$	AWMPFD is external pressure; *E*_i_ is the vulnerability of the *i-*th type of land; *S* is stability.

#### Geographic weighted regression analysis.

The evolution of land conflicts of production, living, and ecological uses in arid inland river basins is influenced by multiple factors. Disparities in topography, geomorphology, and hydrothermal conditions lead to uneven distribution and varying degrees of sensitivity to human disturbances. Natural environmental factors form the basis for the evolution of land conflicts of production, living, and ecological uses. Amidst rapid and sustained economic and social development under urbanization, there is a drastic transformation in territorial element allocation and spatial structure. Socio-economic factors become the direct driving force behind the evolution of land conflicts of production, living, and ecological uses. In light of this, this study is guided by the theory of human-environment regional systems and the theory of social-economic-natural compound ecosystems. Drawing upon relevant research findings and the actual situation in the Shiyang River Basin, the study selects six indicators—elevation, slope, GDP, road network density, population density, and river network density—representing natural, social, and economic aspects. Using the year 2020 as a temporal reference point, an adaptive method is employed to determine weights, with the corrected Akaike Information Criterion (AICC) sample selected to determine the optimal bandwidth [48]. Based on multiple experiments, it has been demonstrated that a 3 km ×  3 km grid scale effectively captures the impacts of various factors on the evolution of land use conflicts in production, living, and ecological areas over a large portion of a watershed. This grid scale strikes a balance by maintaining a relatively low data volume while avoiding the loss of detailed information due to excessively large grids. It also reduces the complexity of data processing and analysis to some extent. Furthermore, the Geographically Weighted Regression (GWR) model takes into account geographical variations, allowing regression coefficients to differ across locations, which enhances its ability to more accurately capture the spatial dynamics of the data. This makes the GWR model particularly advantageous in revealing spatial heterogeneity. Through visualizations such as maps, the GWR model can intuitively display the relationships between variables across different regions, facilitating a deeper understanding of the spatial patterns underlying the data. Such insights contribute to the exploration of spatial relationships between variables. Therefore, this study employs a 3 km ×  3 km grid scale to conduct regression analysis using the GWR model in ArcGIS software. The dependent variable is the comprehensive land use conflict index for the Shiyang River Basin in 2020, while the explanatory variables include elevation, slope, population density, GDP per unit area, road density, and river network density in the same year. The specific formula is as follows:


$$Y_{i}=\beta_{0}\left(u_{i},v_{i}\right)\overset{p}{\underset{k=1}{\sum }}\beta_{k}\left(u_{i},v_{i}\right)x_{ik}+\varepsilon_{i}$$

In the equation, (ui, vi) represents the coordinates of the i-th sampling point, β_0_ is the constant of the model, β_k_ denotes the k regression parameters for the i-th sampling point, ε_*i*_ indicates the residual for the i-th sampling point, and β is a function of the geographical coordinates (u_*i*_, v_*i*_).

If the equation is independent of geographical coordinates, the above formula can be transformed into a standard linear regression. The parameter estimation for each sampling point is associated with a weighted distance matrix constructed by the spatial weight function. Typically, a Gaussian function is employed to construct the weighting function, representing the relationship between spatial weight and spatial distance. The Gaussian function to determine the weight function is as follows [49]:


$$\omega_{ij}=e^{-{\left(\frac{d_{ij}}{b}\right)}^{2}}$$

In the equation, ${\text{ω}}_{\text{ij}}$ represents the spatial weight between sampling point i and sampling point j, d_ij_ denotes the distance between point i and point j, and b is the bandwidth. When the bandwidth b is determined, as the distance d_ij_ increases, the weight assigned to point j decreases, and the weights for points sufficiently far from point i will tend towards 0.

### Result and analysis

#### Analysis of land use pattern evolution characteristics.

**Fig 4:**
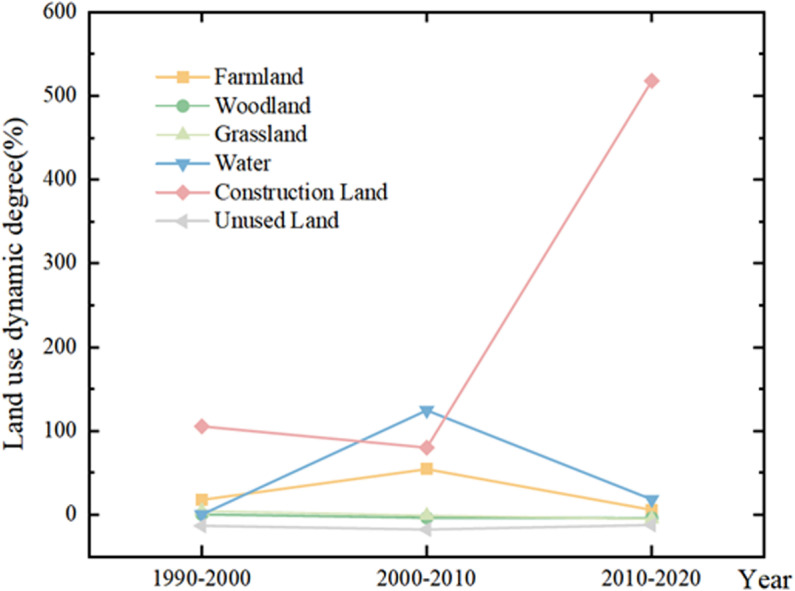
Dynamic Indices of Land Use Types in the Study Area.

**Fig 5:**
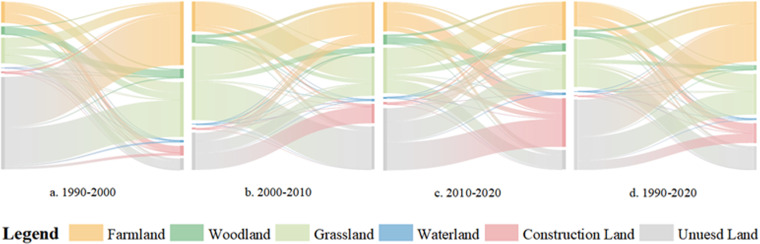
Land Use Change Transfer Diagram.

By calculating the dynamic indices of different land use types in the study area over different time periods (Fig 4), the research reveals a trend in the dynamics of construction land showing a slight decline followed by a significant increase, reaching its peak from 2010 to 2020. This was primarily attributed to a substantial increase in urbanization levels in the Shiyang River Basin during this period, along with the construction of various development zones, industrial parks, and ecological resettlement areas. The dynamics of farmland and water also exhibit pronounced changes, both showing an initial increase followed by a decrease, with the highest dynamics observed between 2000 and 2010. This can be attributed primarily to the peak period of reclamation and cultivation in the Shiyang River Basin during this time frame. Additionally, the Shiyang River Basin experiences a predominantly arid climate with low precipitation, leading to a reliance on irrigation for agricultural activities. Consequently, a substantial number of water resource infrastructure projects were implemented during this period, contributing to significant fluctuations in the dynamism of both farmland and water areas. Based on the land use transfer matrix model analyzing the evolution characteristics of land use patterns, it is evident from the Fig 5 that during the period from 1990 to 2000, the area of unused land underwent the greatest change, decreasing by 237.1 km^2^. Unused land transitioned to farmland and construction land by 189.41 km^2^ and 49.47 km^2^ respectively. This was mainly due to the increased population leading to higher demand for food and residential space, prompting the large-scale development of undeveloped desert areas into farmland and construction land. During the period from 2000 to 2010, the farmland and water body areas in the Shiyang River basin increased by 405 km^2^ and 17.40 km^2^ respectively, while unused land decreased by 317.46 km^2^. This was primarily to support the significant increase in food production and rapid industrialization, facilitated by the construction of modern irrigation networks in the Hexi Corridor, which rapidly enhanced the region’s water resources and food security capabilities. From 2010 to 2020, the largest change was observed in construction land, which increased by a total of 231.33 km^2^. This period marked the peak of construction and development in the Shiyang River basin, primarily driven by urban expansion in areas such as Liangzhou District and Jinchuan District, as well as the implementation of a series of ecological migration and geological disaster avoidance resettlement projects, which further accelerated urban development and construction.

#### Analysis of evolution characteristics of production-living-ecology land.

**Fig 6:**
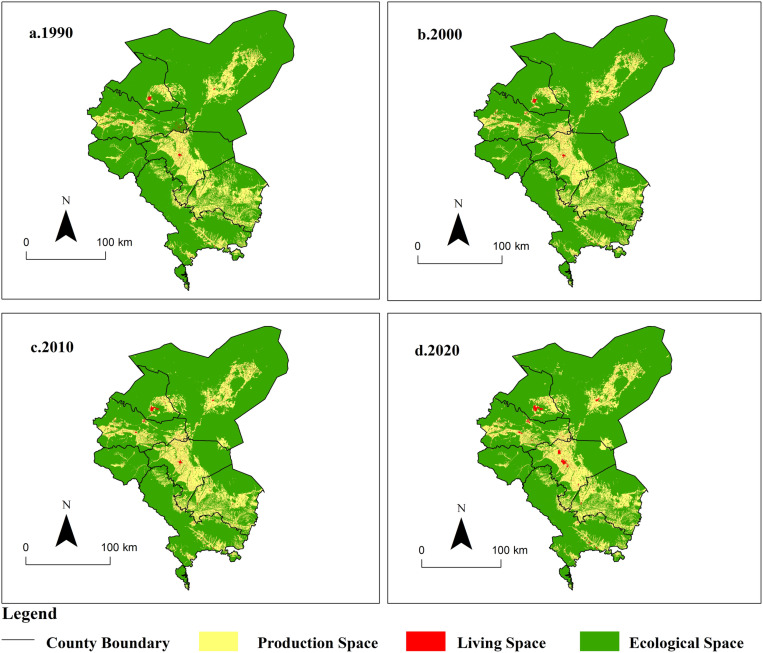
Spatial Distribution Pattern of Production-Living-Ecological Land in Shiyang River Basin from 1990 to 2020. (The map was generated by ArcGIS 10.4 http://www.esri.com/software/arcgis and does not require any permission from anywhere).

**Fig 7:**
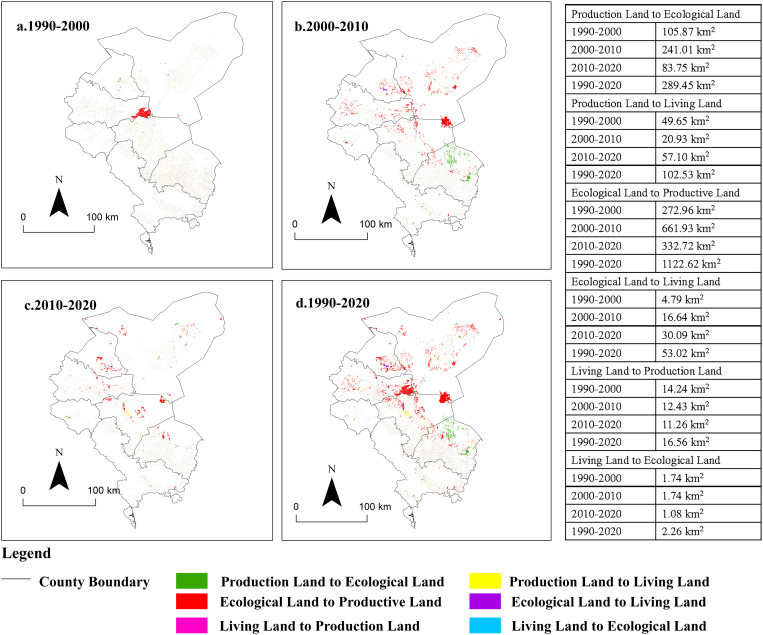
Production-Living-Ecological Land Transformation in Shiyang River Basin from 1990 to 2020. (The map was generated by ArcGIS 10.4 http://www.esri.com/software/arcgis and does not require any permission from anywhere).

According to the classification criteria proposed in this paper for production, living, and ecological land, the layout maps of these land types in the Shiyang River Basin for the years 1990, 2000, 2010, and 2020 were generated (Fig 6). The area of living land increased from 338.81 km² in 1990 to 475.54 km² in 2020, accounting for only 1.12% of the total area of the Shiyang River Basin in 2020. This is primarily attributed to the limited land carrying capacity in arid inland river basins and the sparse distribution of population. Rural residential areas are scattered throughout the central oasis area along the Shiyang River Basin, while urban living space is concentrated mainly in Jinchuan District and Liangzhou District. The area of production land increased from 7,240.72 km² in 1990 to 7,987.92 km² in 2020, accounting for 18.81% of the total basin area in 2020. The Shiyang River Basin is predominantly comprised of agricultural production land, with relatively less industrial and mining production land, primarily situated in the central region of the basin, in the intermediate zone between ecological and living land. The area of ecological land decreased from 34,894.6 km² in 1990 to 34,010.6 km² in 2020, with the proportion of living land in the basin declining from 82.15% in 1990 to 80.07% in 2020, closely correlated with human production and habitation. Although ecological land constitutes a significant portion of the basin, it mainly comprises desert and Gobi composite ecosystems, characterized by overall weak ecological system service functions. Meanwhile, employing the transfer matrix model, the structural transformation of production, living, and ecological land across different stages of the Shiyang River Basin was examined, and spatial visualization was conducted (Fig 7). It is evident that from 1990 to 2020, the structural transformation of production, living, and ecological land in the Shiyang River Basin primarily involved mutual conversions between production and ecological land, followed by mutual conversions between production and living land, with the least extent of conversion observed between land and ecological land. The most significant transformation observed from ecological to production land during 1990-2020 was largely attributed to the extensive conversion of ecological land in the agricultural-pastoral transitional zone into farmland, leading to the gradual encroachment of human agricultural activities into desert areas. Areas with the most substantial conversion were predominantly located in Nanhu Town of Minqin County and Zhuwangpu Town of Yongchang County. The conversion from production land to ecological land was mainly concentrated in Gulang County and Minqin County, associated with initiatives such as returning farmland to woodland and grassland, and windbreak and sand fixation measures, exemplified by sites like the Babusha Forest Farm. Production and ecological land were transformed into living land by 102.53 km² and 53.02 km², respectively, primarily due to rapid urbanization and the expansion of urban construction, encroaching upon ecological and cultivated land.

#### Analysis of production-living-ecological land conflicts.

Analysis of Production-Living- Ecological Land Conflict Indicators

**Fig 8:**
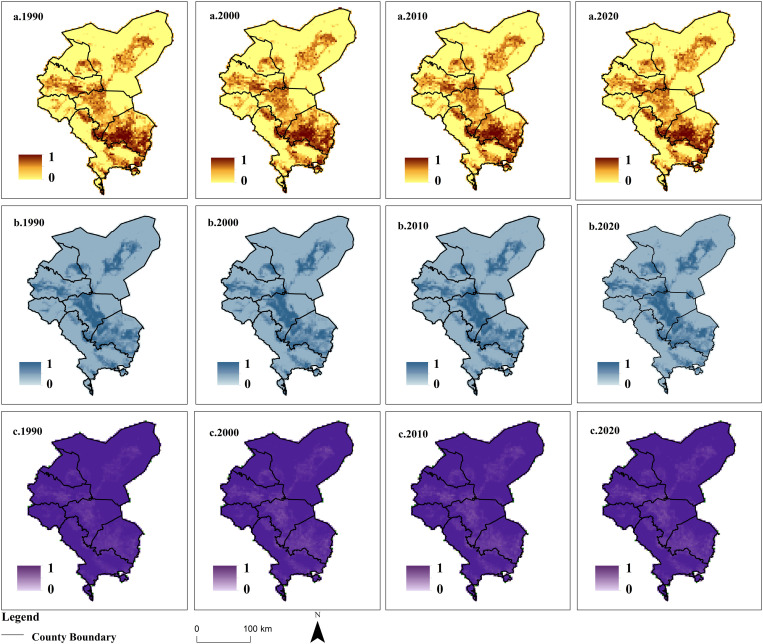
Spatial Distribution of Land Use Complexity, Vulnerability, and Stability in the Shiyang River Basin from 1990 to 2020. **(The map was generated by ArcGIS 10.4 http://www.esri.com/software/arcgis and does not require any permission from anywhere).** a. Spatial Complexity Index b.Spatial Vulnerability Index c.Spatial Stability Index.

Based on the conceptual model of “the risk source—the risk receptor—the risk effect”, this paper constructs an index system comprising three indicators: complexity, fragility, and stability. Visualization of these indicators (Fig 8) is conducted to reflect the characteristics of land use conflicts in the Shiyang River Basin related to production, living, and ecology. Regarding land use complexity, the overall pattern in the Shiyang River Basin exhibits a “high in the south, low in the north” trend, indicating greater interference between production, living, and ecological land in the southern region. This is mainly due to the southern region being the primary agglomeration area for population and industries in the Shiyang River Basin. The southern part of Gulang County shows the highest land use complexity, mainly attributed to relatively better water and soil resources and a higher population carrying capacity, resulting in diverse functions in the territory space. From the perspective of land use vulnerability, the overall land use vulnerability pattern in the Shiyang River Basin follows a “high in the central region, low in the surrounding areas” trend, which aligns closely with the pattern of production and living land. Liangzhou District exhibits the highest land use vulnerability, primarily because it is one of the areas with the highest population and economic density in the inland river basin of the arid region. The overall land use stability in the Shiyang River Basin is relatively high, mainly due to the vast land and sparse population in the arid inland river basin. The patch density is generally small. Particularly for widely distributed mountain and desert ecosystems, human production and living activities are limited, resulting in higher land use stability.

#### Analysis of production-living-ecological land conflict intensity.

**Fig 9:**
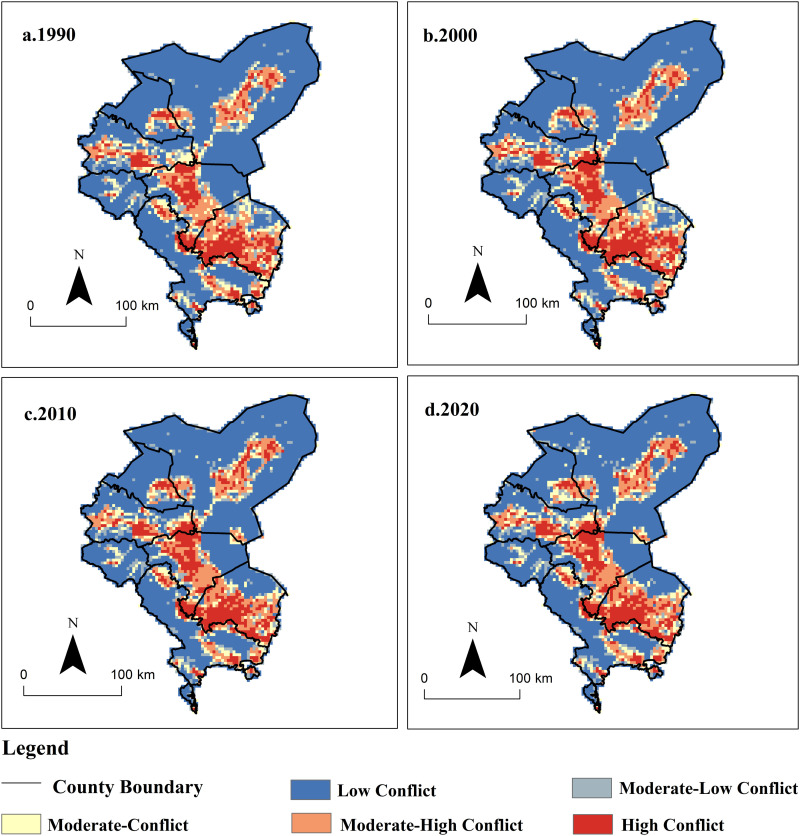
Land Use conflict pattern characteristics of production-living-ecological Land in Shiyang River Basin from 1990 to 2020. (The map was generated by ArcGIS 10.4 http://www.esri.com/software/arcgis and does not require any permission from anywhere).

Based on the comprehensive land use conflict index model, the comprehensive situation of production-living-ecological space conflict in the Shiyang River Basin at different stages was obtained, and the land use characteristics of conflict levels were visualized (Fig 9). From 1990 to 2020, the intensity of production-living-ecological land conflicts in the Shiyang River Basin showed a certain upward trend, with the average conflict level increasing from 0.44 to 0.57. As indicated in Table 3, high conflict and moderate-high conflict areas increased by 990 km^2^ during the study period, accounting for the proportion of the basin’s total area rising from 21.4% to 23.6%, mainly distributed in the central oasis area of the basin. Simultaneously, with the rapid advancement of urbanization, areas with moderate-high and high land use conflicts gradually spread to surrounding regions, increasing in concentration, transitioning from scattered point-like distribution to clustered spatial distribution. This trend is mainly influenced by factors such as urbanization and agricultural land development. From 1990 to 2020, areas with moderate-low and low land use conflicts predominated in the Shiyang River Basin, accounting for over 67% of the total. The proportion of moderate-low and low land use conflicts howed a fluctuating downward trend, decreasing by 945 km^2^ during the study period, reducing from 69.4% to 67.3% of the basin’s total area. These types are mainly distributed in the Ushock Ridge mountainous area and the periphery of the Tengger Desert, characterized by forest and grassland ecosystems. The proportion of moderate land use conflicts howed a fluctuating downward trend, decreasing from 9.18% in 1990 to 8.64% in 2020, primarily distributed in the periphery of production and living land. Overall, from 1990 to 2020, the land use conflict level in the Shiyang River Basin exhibited a pattern characterized by “higher in the central area and lower in the surrounding regions.” This is mainly due to the basin being a typical mountain-oasis-desert composite ecosystem, where the central oasis area serves as the main human production and living aggregation area. Influenced by factors such as urban expansion, agricultural land development, and industrial development, the conflict level in the central area increased. In contrast, the peripheral regions outside the oasis consist of mountain and desert ecosystems, where the implementation of policies such as reforestation, ecological migration, and protection measures has kept land use conflicts at a relatively low level.

**Table 3:** Distribution area of land use conflict levels among production, living, and ecological land.

Type\Year	1990	2000	2010	2020
Area of low conflict (km^2^)	27297.23	26748.54	26775.25	26505.55
Area of moderate-low conflict (km^2^)	3933.35	4311.21	3906.47	3789.18
Area of moderate conflict (km^2^)	4131.68	3879.45	3888.19	4077.20
Area of moderate-high conflict (km^2^)	5301.25	5076.58	5400.26	5490.09
Area of high conflict (km^2^)	4329.28	4977.01	5022.62	5130.77

#### Analysis of Factors Influencing the Evolution of Production-Living-Ecological Land Conflicts.

**Fig 10:**
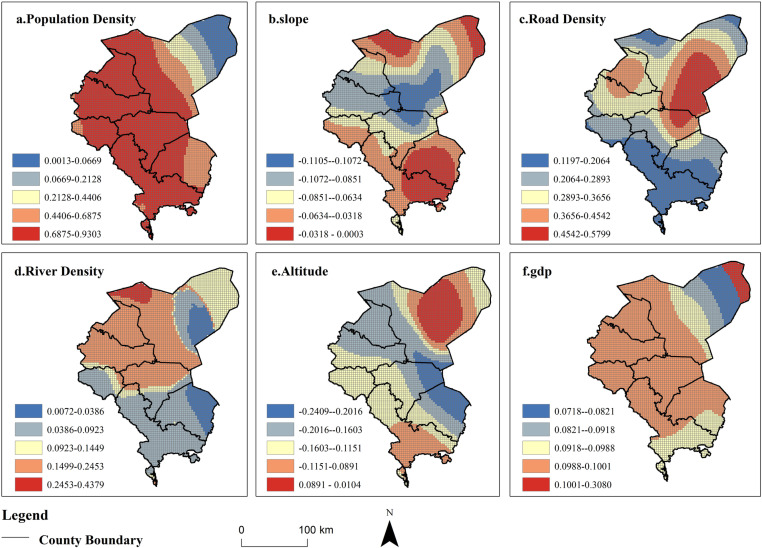
Spatial distribution of regression coefficients of influencing factors of GWR model. (The map was generated by ArcGIS 10.4 http://www.esri.com/software/arcgis and does not require any permission from anywhere).

This study conducted a global spatial autocorrelation analysis on the comprehensive index of conflicts among production, living, and ecological land. The results revealed a Moran’s I index of 0.8657, which was significant at the 0.01 level, indicating a strong spatial autocorrelation feature of the conflict comprehensive index in the study area. Therefore, a Geographically Weighted Regression (GWR) model was employed to explore the influencing factors. The spatial distribution maps of regression coefficients between the conflict index and influencing factors revealed the following trends (Fig 10): ① Population density was positively correlated with production, living, and ecological land conflicts. In regions with higher population density, the comprehensive conflict index might be higher. As urbanization advances, in the oasis of arid areas with limited land resources, higher population density leads to stronger human activity penetration and impact on production, living, and ecological land. This increased demand for production and living land, in turn, compresses the stock of ecological space, intensifying production, living, and ecological land conflicts. ② Elevation and slope were negatively correlated with production, living, and ecological land conflicts. Higher elevation and slope values were associated with lower comprehensive conflict index values. The central area had the largest absolute value of regression coefficients, mainly due to the significant restriction of complex terrain conditions on human activities. The relatively flat terrain in the central region allowed it to dominate in terms of production and living land. Road density was positively correlated with space conflicts. Regions with higher road density values had higher comprehensive conflict index values. ③ Road density reflects the degree of human activity’s impact on natural ecosystems. Areas with higher road density experience greater flow of people, goods, and information, leading to more intense human production and living activities, thus exacerbating production, living, and ecological land conflicts. Spatially, areas closer to the city center with higher road density resulted in higher conflict index values near the junction of Jinchang City and Wuwei City. ④ Per capita GDP was positively correlated with production, living, and ecological land conflicts. Higher per capita GDP in a region was associated with a higher comprehensive conflict index. Economic development and urbanization often lead to the large-scale expansion of urban living and industrial production land. The continuous increase in demand for food also leads to extensive cultivation of farmland, thereby driving the expansion of rural production and living land, aggravating the degree of production, living, and ecological land conflicts. ⑤ River network density was positively correlated with production, living, and ecological land conflicts. Regions with higher river network density had higher comprehensive conflict index values. In arid inland river basins, human production and living depend mainly on glacial meltwater, with rivers being the primary water supply source. Irrigation systems formed by rivers are crucial for agricultural production, and thus, areas closer to rivers experience more intensive human activities, resulting in higher comprehensive conflict index values for production, living, and ecological land conflicts.

#### Territory spatial Optimization Pattern and Regulation Strategies in the Shiyang River Basin.

Territory spatial Optimization Pattern in the Shiyang River Basin

**Fig 11:**
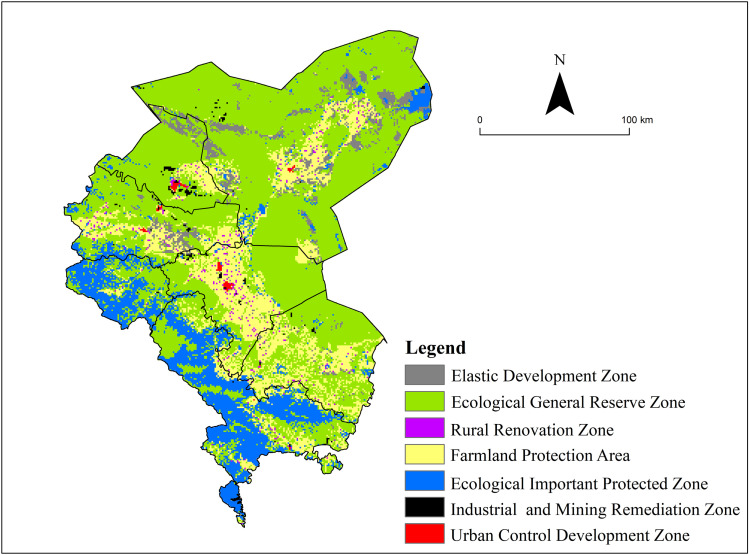
Spatial optimization pattern of land in Shiyang River basin. (The map was generated by ArcGIS 10.4 http://www.esri.com/software/arcgis and does not require any permission from anywhere).

Utilizing a research framework encompassing spatial identification, conflict recognition, mechanism exploration, and rigid constraints, the territory spatial optimization pattern in the Shiyang River Basin was derived (Fig 11). The areas, ranked by percentage of total land area from largest to smallest, include General Ecological Protection Zone (60.88%), Farmland Protection Zone (18.24%), Extremely Important Ecological Zone (14.26%), Flexible Development Zone (5.00%), Urban Control and Development Zone (0.90%), Industrial and Mining Rehabilitation Zone (0.48%), and Rural Renovation Zone (0.24%). The Extremely Important Ecological Zone is predominantly situated in the southwest, encompassing Tianzhu and Sunan counties. This region, anchored by the Qilian Mountains National Forest Park, exhibits the strongest ecological system services and water conservation capabilities in the Shiyang River Basin. The Urban Control and Development Zone is mainly composed of towns such as Liangzhou, Jinchuan, Minqin, Gulang, and Yongchang, serving as the primary population and industry-bearing area in the basin. The Rural Renovation Zone is scattered across the oasis area in the central part of the basin and requires further optimization of the rural living environment. The Flexible Development Zone is primarily located in the northern edge of the oasis area, serving as a potential space for ecological construction, land development, and new energy bases in the future. The Industrial and Mining Rehabilitation Zone is distributed in various areas of Jinchang City, mainly due to its importance as a significant base for non-ferrous metal extraction and smelting in China. The Farmland Protection Zone is situated in the central oasis region, with a higher concentration in Minqin County and Liangzhou District, relying on the irrigation network of the Shiyang River and serving as a crucial area for China’s grain and vegetable production. The General Ecological Protection Zone is positioned in the northern part of the basin, mainly distributed in the western regions of the Badain Jaran Desert and the Tengger Desert, dominated by grassland, desert, and Gobi ecosystems.

#### Spatial Regulation Strategies for the Shiyang River Basin.

Regulating the spatial patterns of land in arid inland river basins is a strategic objective of global and long-term significance. Tailoring differentiated spatial regulation strategies for different spatial patterns can provide policy support for alleviating ecological environmental risks in the Shiyang River Basin and achieving sustainable land use. Ecological restoration and protection areas encompass extremely important ecological protection areas and general ecological protection areas. The level of land-use conflict in critically important ecological protection zones is relatively low, and human activities such as public service facilities, construction, and industrial or mining developments should be strictly prohibited in these areas. Residential area migration should be guided reasonably and orderly. Strengthening the protection of wetlands, forests, and natural reserves, facilitating wildlife migration through “point-line-plane” ecological corridors, and enhancing the connectivity of ecological corridors are essential. The level of land-use conflict in general ecological protection zones is relatively low. It is imperative to vigorously implement ecological restoration and governance projects, such as the protection and restoration of shelterbelts and natural forests, the conversion of cropland to forest and grassland, and the ecological restoration of mining areas. Additionally, further refinement of the list of industries and projects permitted, restricted, and prohibited in these zones is necessary. Production and development protection zones mainly include farmland protection zones and industrial and mining restoration zones. Farmland protection zones should strengthen comprehensive land consolidation, further optimize the layout of agricultural production, control the scale of cultivated land, optimize existing irrigation systems, reduce water supply pressure, develop intensive and efficient water-saving green agriculture. The Shiyang River Basin is mainly dominated by industries related to non-ferrous metal mining and smelting, fine chemicals, new materials, and new energy. Therefore, efforts should be made to accelerate green mining, actively restore mines, promote land disposal, rectify enterprises with high consumption and low efficiency. These actions will significantly enhance the economic output of industrial land, optimize the structural distribution of industries, and reduce spatial conflicts within the region’s territory.

The level of land-use spatial conflict in areas designated for residential and environmental rehabilitation is relatively high, primarily encompassing urban-controlled development zones and rural revitalization areas. Urban development control areas should focus on the efficient utilization of construction land and the optimization of industrial, population, and spatial structures [50]. To improve the efficiency of spatial resource allocation, it is imperative to delineate urban development boundaries and regulate the scale of urban expansion. This involves the restoration of urban ecosystems and the establishment of urban green infrastructure. Leveraging urban landscape patterns, wetland systems, and green corridor networks is essential for constructing green infrastructure, thereby restoring the balance between artificial and natural environments. In rural regulation zones, it is crucial to enhance ecological compensation mechanisms and ecological relocation systems. This includes expediting the systematic relocation of rural settlements in ecologically fragile and significant areas, strengthening control over human activities in these regions, and designating concentrated and intensive rural residential areas to minimize fragmentation. Furthermore, efforts should be made to improve the efficiency of rural land use, prevent the phenomenon of “building new without dismantling old,” and implement rural greening and beautification initiatives. Flexible development zones facing ecological challenges such as climate change, environmental degradation, and biodiversity loss should be prioritized for adjustment into ecological protection flex zones. In the event of droughts, floods, and decreased agricultural productivity, these zones can be adjusted into agricultural production flex zones. Similarly, when confronted with shortages of construction land due to industrial development, urbanization, ecological migration, etc., they can be reorganized into industrial production flex zones and urban living flex zones.

## Discussion

### Discussion on the primary types of land use conflicts in the shiyang river Basin

Land use conflict research originated from the analysis of urban-rural contradictions arising from the conversion of agricultural land to non-agricultural land in the process of urban spatial development. Building upon previous research, Zhou De et al13. employed a comprehensive land use conflicts index to quantitatively evaluate the level of land use conflicts, contributing to the maturity of land use conflicts research [51]. This study analyzes the main types of land use conflicts in the Shiyang River Basin based on the results of land-use changes and the assessment of production, living, and ecological land conflicts. The research reveals that the most significant land use conflicts type in the study area from 1990 to 2020 is the conflict between ecological and production land. Land-use change analysis also indicates a significant transformation between ecological and production land during this period. The Shiyang River Basin is primarily characterized by ecological land, situated in the transitional zone between the Qilian Mountains and the Badain Jaran Desert. The ecological environment quality is significantly influenced by the spatial distribution of water resources [52]. With the development of urbanization, the proportion of water used for production and living significantly increases, encroaching on ecological water within the natural watershed. This leads to regional degradation of ecological environmental quality, decreased vegetation coverage, and a reduction in the area of ecological land. Simultaneously, to meet the rapid economic growth of the region, many developing countries pursue economic growth through agricultural expansion. In many inland river basins, the expansion of cultivated land is a significant factor leading to a decrease in the proportion of ecological land. Particularly in the grassland areas where agriculture and animal husbandry intersect, the conversion of grassland and unused land into cultivated land increases the area of production land, resulting in a higher level of land use conflicts. Therefore, strict control over the expansion of cultivated land in inland river basins is of great significance for maintaining regional ecological security and protecting biodiversity.

### Discussion on the evolutionary process of land use conflicts in the shiyang river Basin

The generation and disappearance of things follow objective development laws, and the coordination process of land use conflicts is also a process of regional development [53]. land use conflicts is the game process of various land types occupying limited land resources. The land use conflicts in a particular region will gradually stabilize under the drive of specific influencing factors in the development of the game process unless new driving factors cause the landscape pattern to evolve in the opposite direction. The evolution of land use conflicts is influenced by the initial landscape pattern in the early stages of the study, and different land use conflicts change patterns are presented in different landscape change processes. This study employs geographic weighted regression to analyze the influencing factors of the evolutionary process of land use conflicts in the study area. The results indicate that road network density and river network density have the most significant impact on the production, living, and ecological land conflicts in the Shiyang River Basin. Road network density reflects the accessibility of the region, especially for the inland Shiyang River Basin. Road construction accelerates the circulation of human, material, economic, and information flow, greatly promoting the development of urbanization and significantly influencing the evolutionary process of land use conflicts. River network density is determined by regional precipitation, altitude, and soil water retention capacity. The Shiyang River Basin has low precipitation, a west-high and east-low altitude, insufficient water retention capacity, and is downstream of the Badain Jaran Desert. Human production and living water are mainly concentrated in the middle reaches, resulting in land use conflicts mainly occurring in the central region. Therefore, the river network is a crucial factor influencing the evolution of land use conflicts. In conjunction with the actual situation in the study area, urbanization, agricultural land expansion, and desertification are the main driving forces for changes in production, living, and ecological land in the Shiyang River Basin, also the primary factors leading to changes in land use conflicts [54,55]. The expansion of urban space is mainly characterized by edge growth, and the expansion of urban space changes the landscape pattern embedded with living and production land in the transition zone between urban and rural areas. The spatial heterogeneity of the landscape is reduced, and the level of land use conflicts decreases. With the continuous expansion of urban edges, the originally complex and heterogeneous landscape pattern becomes homogeneous and single. If not influenced by policies or other natural factors, the level of land use conflicts will gradually stabilize. However, a scenario different from edge expansion exists, where the expansion of urban areas within other land uses far from urban areas will lead to an increase in the level of land use conflicts. For example, constructing villas, factories, etc., in suburban areas disrupts the originally homogeneous landscape, enhances spatial complexity, and causes an increase in the regional level of land use conflicts. With the expansion of production and living land in the region, the level of land use conflicts will initially increase and then decrease, eventually stabilizing.

### Exploration of the mechanisms behind territory spacial conflicts in inland river Basins

**Fig 12:**
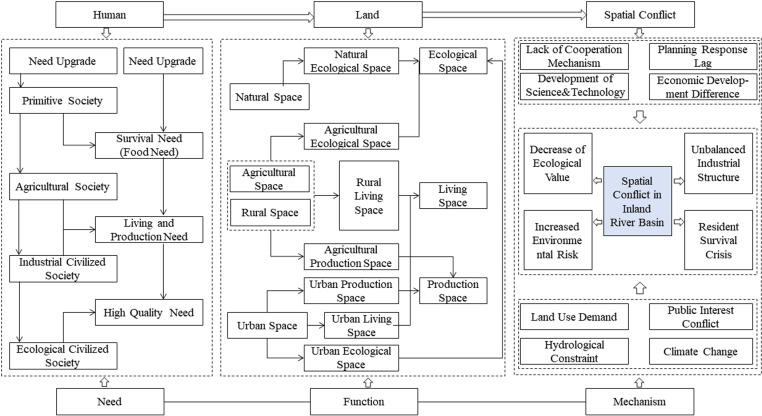
Mechanism of land-use conflict in inland river basin.

Examining the history of human civilization, the relationship between humans and the environment at different times influences the temporal sequence, functions, scale, structure, and layout of spatial development, thereby shaping the evolution of national territory spatial patterns. Due to differences in natural conditions, resource foundations, development histories, population densities, and other factors among different regions, the manifestation, intensity, and feedback relationships of human-environment relationships exhibit significant regional variations. This determines diverse spatial structures in different regions, shaping distinct configurations of production, living, and ecological land and influencing the evolution of land use conflicts. Therefore, this discussion aims to interpret the mechanisms behind the formation of land use conflicts in inland river basins based on the perspective of production, living, and ecological considerations, grounded in the developmental stages of human civilization and the evolution of human-environment relationships (Fig 12). The occurrence mechanisms of land use conflicts in inland river basins are complex and involve various factors such as resource utilization, environmental protection, and socio-economic development [56]. The abundance of water resources plays a decisive role in the production and living aspects of inland river basins. When different interest groups compete for limited water resources, it may lead to over-exploitation and competition for water resources, triggering conflicts in land use. Conflicts may arise due to conflicting demands for land use among different industries, regions, and social groups. Different goals in land use, such as agriculture, urban construction, industry, and ecological protection, combined with a lack of effective cooperation mechanisms, can lead to issues of resource use imbalance and competition, resulting in conflicts in land use. High economic primacy in widespread areas of inland river basins creates significant disparities in regional socio-economic development levels. Imbalances in resource allocation and utilization among regions, coupled with conflicting interests between regions, lead to conflicts in land use. Different government agencies at various levels may have different policies and regulations, and a lack of coordination may lead to contradictions in resource use and territory spatial planning. Implementation of policies may face issues of delay and inconsistency. In the process of national territory spatial planning and decision-making, insufficient public participation or inadequate transparency in information may lead to contradictions and conflicts among different interest groups. Inland river basins may be influenced by climate change, resulting in the instability of water resources. Natural disasters such as floods and droughts may exacerbate conflicts over resources and land use [57].

### Expectation

Acknowledging the limitations inherent in this study, there remain three key areas for advancement in subsequent research: ①This study constructs a land-use conflict intensity identification system based on the conceptual model of “the risk source—the risk receptor—the risk effect”. It solely employs a land-use conflict index to represent the static intensity, lacking attributes such as the rate and direction of conflict changes. Thus, further supplementation and refinement are needed to predict the future risk trends of land-use conflicts. ②The evolution of production, living, and ecological land conflicts is influenced by multiple factors. These factors affecting their evolutionary paths are not limited solely to economic, social, and natural aspects. Subsequent research in this area requires further enhancement. ③Land-use conflicts exhibit characteristics of complexity and dynamism, impacting regional land spatial patterns and security. Subsequent efforts should focus on predicting land use conflict simulations to optimize national land spatial patterns, facilitating multi-objective collaborative research aimed at ecological conservation, food security, and economic growth. These endeavors are in dire need of strengthening.

## Conclusion

(1) The findings indicate that the unused land area in the Shiyang River Basin decreased significantly from 1990 to 2020, mainly converted to farmland, construction land, and water. The transformation from ecological to production land reached 1122.62 km^2^ during this period, closely related to wasteland reclamation and industrial construction.
(2) The structural transformation of production, living, and ecological land primarily manifested as mutual conversions between production and ecological land, followed by conversions between production and living land, with the smallest area for conversions between living and ecological land.
(3) The overall spatial complexity in the Shiyang River Basin exhibited a “high in the south and low in the north” pattern, while spatial vulnerability displayed a “high in the central area and low in the surrounding regions” pattern. Overall, land use stability was relatively high. The intensity of conflicts in the production-living-ecological land of the Shiyang River Basin showed a certain increasing trend from 1990 to 2020, with the average conflict level rising from 0.44 to 0.57. During the study period, high conflict and moderate-high conflict areas increased by a total of 990 km^2^, mainly distributed in the central oasis area, resulting in a pattern where conflict levels are high in the central area and low in the surrounding regions. This pattern is closely related to the Shiyang River Basin being a composite ecosystem of mountains, oasis, and desert.
(4) Through an analysis of the influencing factors of the evolution of conflicts in the production-living-ecological land, it was found that road density and river network density had the most significant impact in the Shiyang River Basin.
(5) The Shiyang River Basin’s territory space was divided into ecological general protection areas, farmland protection areas, ecologically extremely important areas, resilient development areas, urban control and development areas, industrial and mining restoration areas, and rural transformation areas. Among these, the ecological general protection areas had the largest area, while the rural transformation areas had the smallest area. Regulation strategies were proposed for ecological restoration and protection areas, production and development protection areas, living regulation and protection areas, and resilient development areas. These recommendations aim to provide a scientific basis for the optimization and comprehensive governance of territory spatial patterns in arid inland river basins.
